# Effects of Acute Aerobic Exercise on Sequentially Administered Cognitive Tasks Reflecting Working Memory Subcomponents and Heart Rate Variability

**DOI:** 10.3390/jintelligence14100269

**Published:** 2026-10-17

**Authors:** Melike Esentaş Deveci, Dilan Futsi, Cemal Faruk Rize, Erdem Uylas, Nurten Dinç, Erkan Günay

**Affiliations:** 1 Department of Recreation, Faculty of Sport Sciences, Manisa Celal Bayar University, 45040 Manisa, Türkiye; melike.esentas@cbu.edu.tr; 2 Department of Recreation, Faculty of Sport Sciences, Istanbul Gelişim University, 34310 Istanbul, Türkiye; dilanfutsii@gmail.com; 3 Department of Physical Education and Sports, Institute of Postgraduate Studies, Manisa Celal Bayar University, 45040 Manisa, Türkiye; cemalfrize@gmail.com; 4 Independent Researcher, 45040 Manisa, Türkiye; erdemuylas1995@gmail.com; 5 Department of Coaching Education, Faculty of Sport Sciences, Manisa Celal Bayar University, 45040 Manisa, Türkiye; erkan.gunay@cbu.edu.tr

**Keywords:** acute aerobic exercise, sequential cognitive tasks, working memory subcomponents, heart rate variability, executive function, visuospatial construction

## Abstract

Although acute aerobic exercise selectively influences specific subcomponents of working memory, whether such benefits transfer to a subsequent cognitive task engaging a different domain remains unclear. Forty-seven participants completed, in randomized order, an exercise condition (10 min of moderate-intensity aerobic exercise) and a control condition, each immediately followed by a visuospatial construction task (the Equilibrio game) and a 2-Back working memory task, with heart rate variability (HRV) recorded throughout. Completion time on the Equilibrio task was significantly shorter following exercise than following control, *W* = 292.5, *z* = −2.873, *p* = .004, *r_B_* = −0.481. In contrast, no significant between-condition differences emerged on any behavioral parameter of the 2-Back task, including completion time, accuracy, hit rate, discrimination sensitivity (*d*′), and response bias (*c*; all *p*s > 0.05). During the 2-Back task, however, sympathetic indices (SNS Index, Stress Index, LF/HF ratio) were significantly elevated and parasympathetic/vagal indices (RMSSD, SDNN, PNS Index, HF n.u.) were significantly suppressed following exercise, despite unchanged behavior. These findings indicate that exercise-induced cognitive benefit is task-specific and does not extend to a subsequent task engaging a different working memory subcomponent, even as autonomic activation persists. This dissociation challenges general-arousal accounts of exercise–cognition effects and supports a domain-specific, timing-sensitive framework.

## 1. Introduction

The well-established link between physical activity and cognitive function is one of the most prominent current research topics the fields of sports science and neuroscience. In particular, the current literature clearly demonstrates the macroscopic effects of long-term exercise programs on brain plasticity (Chang et al., 2012; Pontifex et al., 2018). However, the neurophysiological and metabolic effects induced by a single session of acute exercise remain a relevant research question. Acute exercise interventions rapidly increase cerebral blood flow, stimulate neurotransmitter release, optimize prefrontal cortex (PFC) activation, and produce an immediate improvement in cognitive performance (Bediz et al., 2016; McMorris, 2021). The momentary changes that arise in this context necessitate an examination of their combined effects on sensory–motor cognition, executive functions, and autonomic responses observed during task performance (Chang et al., 2012; Pontifex et al., 2018).

The neurophysiological mechanisms underlying the cognitive-enhancing effects of acute exercise involve highly complex processes and vary depending on exercise intensity. This process is often explained in the literature by the “Reticular-Activating Hypofrontality” (RAH) theory (Dietrich & Audiffren, 2011). According to the RAH theory, during high-intensity exercise in particular, motor areas, sensory systems, and central autonomic processes consume a significant portion of the brain’s existing resources—which have limited capacity—in order to meet increased metabolic demands. As a result, a state of hypofrontality occurs due to a temporary reduction in metabolic resources; conversely, during mild to moderate acute exercise loads, neural activation in the prefrontal cortex is stimulated, thereby supporting exercise performance (Dietrich & Audiffren, 2011). In addition to this theoretical process, during low- and moderate-intensity exercise, stimulation of the locus ceruleus–norepinephrine (LC-NE) system increases the release of neurochemicals such as dopamine, norepinephrine, and brain-derived neurotrophic factor (BDNF), thereby acutely facilitating synaptic transmission between brain regions (Reycraft et al., 2020). This is grounded in the Inverted-U Hypothesis (Yerkes-Dodson Law), which explains that, due to this increased neurochemical stimulation, the synaptic signal-to-noise ratio is optimized and marked improvements in performance occur in executive function processes (McMorris, 2021). Specifically, increased activation in brain regions such as the dorsolateral prefrontal cortex (DLPFC) and anterior cingulate cortex (ACC) during exercise plays a pivotal role in enhancing executive functions like memory performance, facilitating inhibition against external distractors, and maintaining task engagement (Braver et al., 1997; Owen et al., 2005).

Following exercise, activity across higher neural centers—including the prefrontal cortex, amygdala, and hypothalamus—is transmitted via the vagus nerve to regulate cardiac rhythm (Thayer et al., 2009). Consequently, shifts in these central neural responses closely align with heart rate variability (HRV) dynamics. Through this pathway, the prefrontal cortex’s regulatory capacity under fluctuating cognitive workloads and exercise-induced stress can be non-invasively tracked via HRV as a surrogate of cardiac autonomic function (Shaffer & Ginsberg, 2017; Thayer et al., 2009). Following acute aerobic exercise, an initial withdrawal of parasympathetic activity (vagal tone) and concurrent sympathetic dominance typically transition into a progressive parasympathetic reactivation during recovery (Stanley et al., 2013). Time-domain (e.g., RMSSD, SDNN) and frequency-domain (e.g., LF, HF, LF/HF) HRV metrics recorded during post-exercise cognitive performance directly reflect neurovisceral flexibility during inhibitory control (Shaffer & Ginsberg, 2017; Thayer et al., 2009), serving as widely recognized markers of post-exercise autonomic recovery dynamics.

The existing literature on the acute exercise–cognition paradigm has predominantly relied on experimental designs implementing single-task protocols or repeated administrations of the same cognitive test. However, paradigms evaluating the carryover or sequential effects on complex, successive cognitive tasks remain markedly understudied. In real-world educational and occupational settings, individuals continuously navigate sequentially presented tasks imposing diverse cognitive demands. These ecological realities highlight the necessity of examining executive functions within a broader, multi-component framework. To address this gap, the present study utilizes a sequential design to evaluate the effects of acute exercise across distinct subcomponents of working memory—specifically sensorimotor/visuospatial processing and executive working memory—alongside task-concurrent autonomic nervous system responses. Accordingly, this study employed a randomized, within-subjects crossover design (Wellek & Blettner, 2012) to examine the effects of acute aerobic exercise on sensorimotor/motor–cognitive performance (Equilibrio task), executive working memory capacity (2-Back task), and concurrent autonomic modulation via HRV metrics. We hypothesized that acute aerobic exercise would selectively enhance performance on the sensorimotor/visuospatial construction task administered immediately post-exercise, whereas the subsequent executive working memory task would show no comparable behavioral facilitation despite sustained autonomic activation; we further hypothesized that exercise-induced autonomic changes (indexed via HRV) would persist across both post-exercise tasks irrespective of behavioral outcome.

## 2. Methods

### 2.1. Participants

The study sample comprised 47 healthy volunteer undergraduate students aged 18–29 years (23 males, 24 females; age: 21.04 ± 2.68 years; height: 173.24 ± 8.52 cm; body mass: 69.79 ± 12.01 kg; body mass index [BMI]: 23.03 ± 2.80 kg/m^2^) enrolled in the Faculty of Sports Sciences at Manisa Celal Bayar University. Inclusion criteria required participants to have no prior experience with brain/mind-builder strategy games and to maintain an International Physical Activity Questionnaire (IPAQ) score of at least 3.3 METs. Individuals were excluded if they reported visual impairments, chronic sleep disorders, current use of psychiatric medications known to modulate cognitive function, less than 8 h of sleep on the test day, or acute consumption of alcohol or caffeine prior to testing.

**Figure 1:**
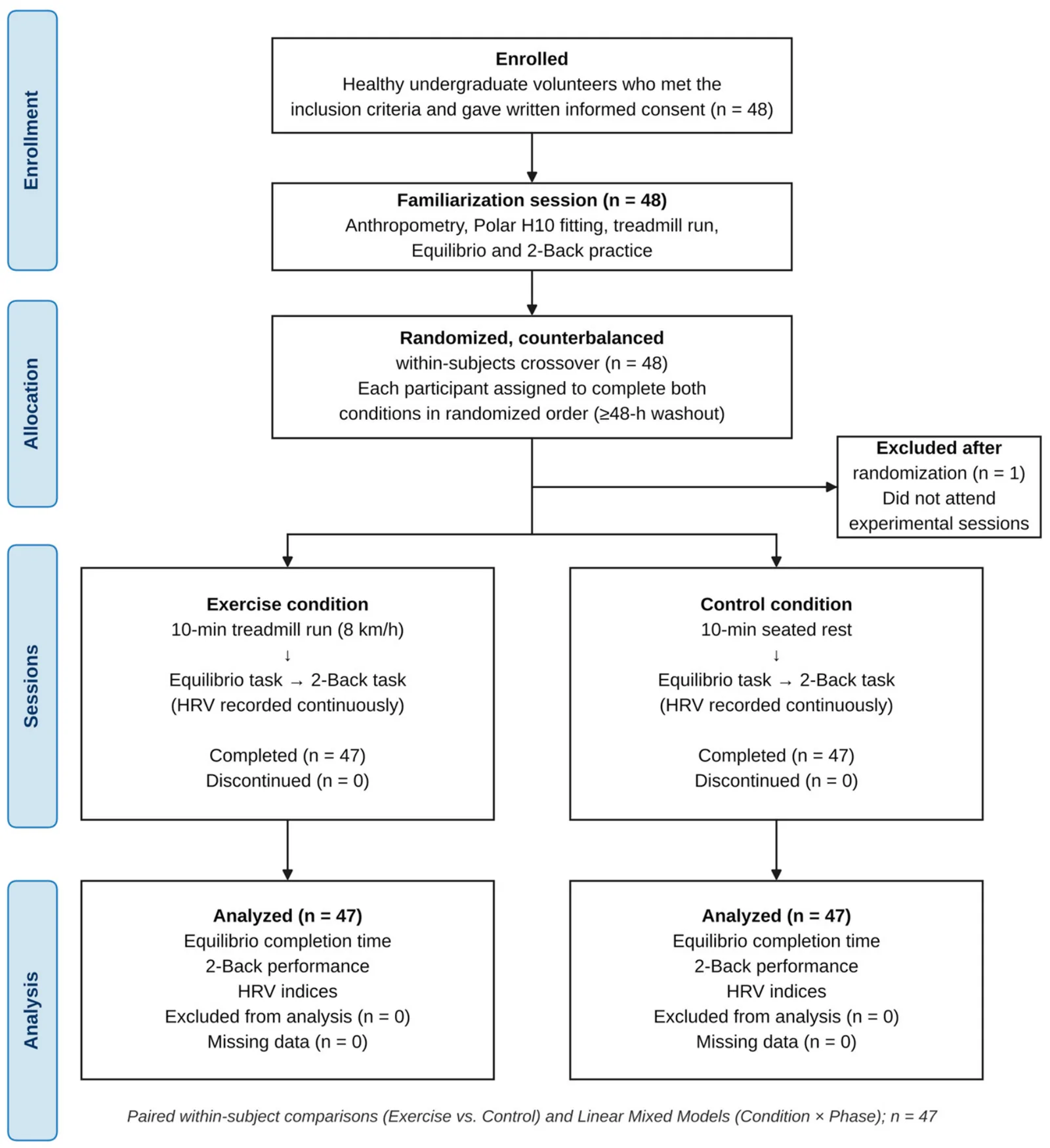
Participant flow diagram. Forty-eight volunteers were enrolled and completed the familiarization session; after randomization to a counterbalanced condition order, one participant was excluded for non-attendance at the experimental sessions. The remaining 47 participants completed both the exercise and control conditions (within-subjects crossover) and were included in all analyses, with no missing data.

An *a priori* statistical power analysis was conducted using G*Power 3.1.9.7 (Faul et al., 2007). Based on a 2 × 2 repeated-measures analysis of variance (ANOVA) design with an effect size of *f* = 0.25 (a medium effect size, consistent with Cohen’s (1988), conventions and with effect sizes reported in the acute exercise-cognition literature; e.g., (Chang et al., 2012), α = 0.05, and statistical power (1 − β) = 0.80, the minimum required sample size was calculated as 34 participants (Kang & Huh, 2021). This a priori calculation was powered to detect the primary paired condition comparisons; the study may nonetheless have been underpowered to detect smaller-magnitude effects or higher-order Condition × Phase interaction effects within the Linear Mixed Models, which typically require larger samples than simple pairwise comparisons. To account for potential data attrition, the study was initiated with 48 participants. One participant was excluded after familiarization for non-attendance at subsequent sessions, yielding a final N of 47. The flow of participants through the study, including enrollment, randomization, completion of both experimental conditions, exclusions, and missing data, is presented in Figure 1. All participants received comprehensive verbal and written briefings regarding the study aims, experimental procedures, and potential risks before providing written informed consent.

The experimental protocol conformed to the ethical principles outlined in the Declaration of Helsinki and was formally approved by the Health Sciences Ethics Committee of Manisa Celal Bayar University (Decision No: 20.478.486/3505, Date: 22 October 2025).

### 2.2. Study Design

This study was structured using a randomized, counterbalanced, within-subjects crossover design (Wellek & Blettner, 2012). Each participant completed three experimental sessions: familiarization, an exercise condition, and a control condition. To rigorously control for potential order and sequence effects, the execution order of the exercise and control conditions was randomized and counterbalanced; participants remained blinded to their allocation sequence until the morning of testing. The within-subjects paradigm allowed each participant to serve as their own control, enabling robust repeated-measures statistical comparisons. Excluding the familiarization phase, each experimental session lasted approximately 20 min, yielding a total testing duration of ~40 min across both experimental arms.

**Figure 2:**
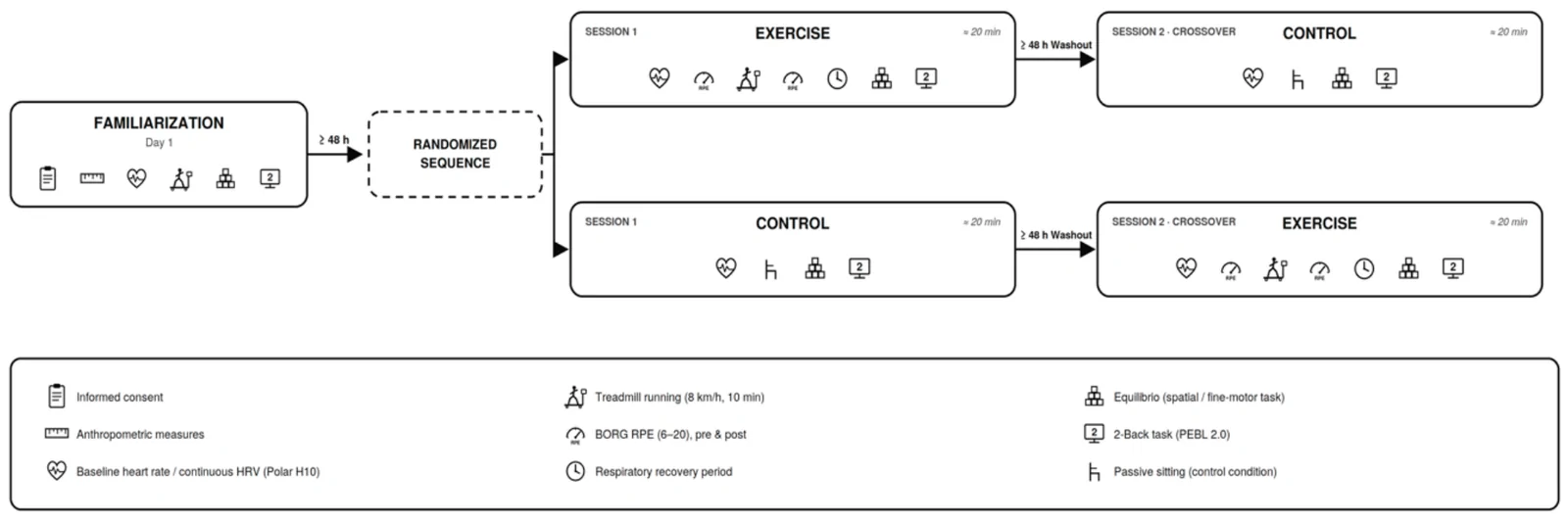
Study Design.

To minimize potential carry-over effects, a minimum 48 h washout period was enforced between sessions (Stanley et al., 2013). All sessions were conducted during the same time window of the day (09:00–17:00) in a quiet, well-illuminated laboratory under standardized environmental conditions (ambient temperature and humidity), utilizing the identical computer workstation. On all testing days, participants were instructed to adhere strictly to the following pre-test guidelines: abstain from smoking, caffeine, and alcohol consumption for at least 2 h prior to testing, and ensure adequate hydration and caloric intake beforehand. The schematic workflow of the experimental design is illustrated in Figure 2.

### 2.3. Measures and Experimental Protocols

#### 2.3.1. Anthropometric Assessments

Participants’ stature was measured in centimeters using a portable manual stadiometer, with individuals barefoot and positioned upright with their backs flat against a wall. Body mass was recorded in kilograms to the nearest decimal using a digital bioimpedance scale (Tanita MC-780, Tanita Corp., Tokyo, Japan) while participants were barefoot and standing erect. Body Mass Index (BMI) was subsequently calculated as body mass divided by height squared (kg/m^2^) (Stewart et al., 2011).

#### 2.3.2. Heart Rate Variability (HRV) Recording and Analysis

Electrocardiographic (ECG) raw signals were captured via a Polar H10 chest strap sensor (Polar Electro Oy, Kempele, Finland) (Gilgen-Ammann et al., 2019). Data acquisition was maintained continuously from the start to the end of each session utilizing the *ECG Recorder for Polar H10* mobile application (v1.6.4; CardioMood Ltd., 2023) and exported as raw .csv files for offline processing. Continuous recordings were segmented into discrete time windows corresponding to the experimental phases to permit factorial comparisons across conditions (exercise vs. control) and time courses (pre- vs. post-intervention). Phase boundaries were determined using a stopwatch initiated simultaneously with the onset of each session, which was used to record the start and end time of every experimental phase (i.e., the initial 10 min intervention/control period, the Equilibrio task, and the 2-Back task); these timestamps were then applied within Kubios HRV software (4.3.0) to segment the continuous ECG recording into phase-specific analysis windows corresponding to the actual duration of each phase.

Raw R–R interval time series were processed using Kubios HRV Premium software (Version 0.97.1, Kubios Oy, Kuopio, Finland) (Tarvainen et al., 2014). Prior to spectral and temporal quantification, Kubios’ automatic artifact correction algorithm was applied using the “medium” correction level, applied uniformly across all experimental phases, to remove ectopic beats and signal artifacts (Lipponen & Tarvainen, 2019); the proportion of corrected artifactual beats averaged below 5% across all experimental phases, which is within commonly accepted data-quality thresholds for ambulatory/exercise HRV recordings. Time-domain metrics included mean heart rate (HR; bpm), the standard deviation of normal-to-normal R–R intervals (SDNN; ms), the root mean square of successive differences between adjacent R–R intervals (RMSSD; ms), natural log-transformed RMSSD (lnRMSSD), and the percentage of adjacent R–R intervals differing by more than 50 ms (pNN50). Frequency-domain power spectral densities were decomposed into Very-Low-Frequency (VLF; 0.0033–0.04 Hz), Low-Frequency (LF; 0.04–0.15 Hz), and High-Frequency (HF; 0.15–0.40 Hz) bands. The LF/HF ratio was computed as an index of sympathovagal balance (Shaffer & Ginsberg, 2017). In addition, Kubios HRV Premium software computed the composite Parasympathetic Nervous System (PNS) and Sympathetic Nervous System (SNS) indices, together with Baevsky’s Stress Index. The Stress Index is derived from the RR-interval histogram (mode, amplitude of mode, and variation range) and reflects the degree of centralization of cardiovascular regulation. The PNS index is calculated from Mean RR interval, RMSSD, and the Poincaré plot SD1 parameter, whereas the SNS index is calculated from Mean HR, the Stress Index, and the Poincaré plot SD2 parameter; each constituent parameter is standardized (z-scored) against normative reference population values and combined via Kubios’ proprietary weighting algorithm (Tarvainen et al., 2014). For both indices, a value of zero indicates activity equivalent to the normative population mean, positive values indicate above-normal activity, and negative values indicate below-normal activity for the respective branch of the autonomic nervous system. Task durations (M ± SD) were 81.36 ± 27.92 s (exercise) and 89.11 ± 23.15 s (control) for Equilibrio, and 237.98 ± 11.27 s (exercise) and 240.62 ± 10.08 s (control) for the 2-Back task; the corresponding mean (±SD) numbers of valid RR intervals per analysis window were 1467 ± 208 (exercise) and 795 ± 67 (control) for the protocol phase, 179 ± 58 (exercise) and 123 ± 31 (control) for Equilibrio, and 451 ± 69 (exercise) and 321 ± 31 (control) for the 2-Back task.

#### 2.3.3. Aerobic Exercise Protocol

During the exercise condition, participants performed a standardized 10 min running bout at a constant speed of 8 km/h on a motorized treadmill (h/p/cosmos saturn, h/p/cosmos sports & medical GmbH, Nussdorf-Traunstein, Germany) (Asimakidis et al., 2025; Berthon et al., 1997). Heart rate was recorded at the onset and cessation of the protocol, and total covered distance was tracked throughout the run.

#### 2.3.4. Rating of Perceived Exertion (Borg RPE Scale)

The 6–20 point Borg Rating of Perceived Exertion (RPE) scale was utilized to assess participants’ subjective perception of physical effort during exercise (Williams, 2017). The scale corresponds to distinct effort classifications: 6–8 denotes very light, 9–13 denotes moderate, 14–17 denotes hard/vigorous, and 18–20 represents maximal exertion. Borg RPE scores were obtained exclusively during the exercise session at two distinct time points: immediately preceding the treadmill protocol and immediately upon its completion (Garber et al., 2011).

#### 2.3.5. Equilibrio Cognitive–Motor Game

To evaluate visuospatial ability and fine motor coordination, the *Equilibrio* board game (Brain Builder Series; FoxMind Games, Montreal, QC, Canada) was administered. Spatial visualization constitutes a core construct within cognitive ability taxonomies, requiring the mental manipulation of three-dimensional objects (Carroll, 1993; Linn & Petersen, 1985). The block-building paradigm underlying *Equilibrio* shares theoretical and operational foundations with classical spatial visualization assessments—most notably the WAIS Block Design subtest—simultaneously recruiting planning, visuospatial reasoning, and fine motor precision (Casey et al., 2012; Valentine et al., 2020). The game consists of a GeoBlock set comprising 18 distinct geometric solid pieces and an instructional manual illustrating 60 three-dimensional structures stratified across six difficulty tiers. Participants were instructed to vertically construct the target models corresponding to the first three sequential levels using the GeoBlocks strictly according to visual blueprints. The dependent outcome measure was defined as the total completion time (in seconds) required to assemble the three initial structural configurations. Completion time was recorded manually via stopwatch by a member of the research team, who administered the task using standardized verbal instructions identical across both conditions. The researcher responsible for timing was blinded to the specific aims of the study, thereby minimizing the risk of experimenter expectancy effects influencing task administration or timing.

#### 2.3.6. 2-Back Task

Working memory and sustained attention performance were evaluated using the letter-based 2-Back task from the Psychology Experiment Building Language (PEBL 2.0) test battery (Mueller & Piper, 2014). The 2-Back paradigm is a standardized cognitive protocol engaging short-term memory, inhibitory control, and memory retrieval processes, known to elicit robust neural recruitment across executive control regions, particularly the dorsolateral prefrontal cortex and anterior cingulate cortex (Bediz et al., 2016; Braver et al., 1997; Owen et al., 2005). A pseudorandomized sequence of 120 uppercase letter stimuli drawn from an 8-character set (K, W, H, X, N, Y, R, C) was presented centrally on a computer monitor, with 40 trials designated as target stimuli. Participants were instructed to determine whether each presented letter matched the one displayed two trials prior. Each stimulus was displayed for a duration of 500 ms, followed by a jittered inter-stimulus interval ranging from 1500 to 2000 ms. Cognitive performance metrics were structured across three primary dimensions: (1) response time dynamics: mean reaction time for correct target detections (Mean RT; ms), standard deviation of reaction times (SDRT; ms), and the intra-individual coefficient of variation (CV = [SDRT/Mean RT] × 100); (2) accuracy and error parameters: target hits, hit rate (%), misses, false alarms, and overall accuracy (%); and (3) Signal Detection Theory (SDT) metrics: perceptual sensitivity index (*d*′ = Z[Hit Rate] − Z[False Alarm Rate]), assessing discriminability of target signals against background noise, and the response bias criterion (*c* = −0.5 × [Z(Hit Rate) + Z(False Alarm Rate)]), reflecting decision-making strategy (Stanislaw & Todorov, 1999). All cognitive assessments were administered on the identical computer workstation under standardized testing conditions.

### 2.4. Experimental Procedure

#### 2.4.1. Familiarization Session

During the initial session, participants received comprehensive information regarding the study protocol and provided written informed consent. Demographic information and anthropometric characteristics (height, body mass, and BMI) were subsequently documented. The primary purpose of this session was to minimize potential learning and practice effects during subsequent testing sessions and to ensure that participants attained operational familiarity with all testing apparatus and procedures (Collie et al., 2003; Hopkins, 2000). In this context, participants were first fitted with the Polar H10 chest strap to perform a brief pilot heart rate recording, followed by an introductory familiarization run on the motorized treadmill. Subsequently, the rules of the *Equilibrio* board game were demonstrated using a structural model to establish task familiarity, and the operational guidelines of the computer-based 2-Back task were introduced and practiced once to ensure task proficiency.

#### 2.4.2. Exercise Session

Upon arrival at the laboratory for the exercise session, participants were fitted with the Polar H10 chest strap, and baseline heart rate recording was initiated. Immediately prior to the treadmill running protocol, participants’ baseline Borg RPE scores were documented. Participants then performed a 10 min treadmill run at a constant speed of 8 km/h. Immediately following the cessation of exercise, a post-exercise Borg RPE score was obtained, and participants were provided a brief 20–30 s respiratory stabilization period. Following this brief recovery phase, participants were seated at the computer workstation to sequentially complete the first three levels of the *Equilibrio* task and the 2-Back working memory task. Heart rate dynamics were recorded continuously throughout the entire experimental session.

#### 2.4.3. Control Session

In the control session, participants were fitted with the Polar H10 chest strap upon arrival to initiate baseline heart rate acquisition and were subsequently instructed to remain seated quietly in a passive state for 10 min. Participants received standardized verbal instructions to remain seated and were not permitted access to mobile phones, reading materials, or any other devices or objects during this period, thereby minimizing uncontrolled cognitive or affective stimulation during the resting interval. This 10 min passive resting period served as a time-matched control condition corresponding to the treadmill running protocol in the exercise arm (Chang et al., 2012; Pontifex et al., 2018), thereby ensuring equivalence in total assessment duration between conditions. Following the resting interval, participants immediately transitioned to the computer workstation to sequentially perform the first three levels of the *Equilibrio* task and the 2-Back task. Borg RPE was not assessed during the control session; however, continuous heart rate recordings were maintained throughout the entire protocol.

### 2.5. Statistical Analysis

Statistical analyses were performed using JASP software (Version 0.97.1) (JASP Team, 2026). The assumption of normality was evaluated for all continuous variables using the Shapiro–Wilk test (Field, 2018). For normally distributed paired observations across the two experimental conditions, paired-samples *t*-tests were conducted, with effect sizes quantified using Cohen’s *d* (Cohen, 1988). For non-normally distributed parameters, paired comparisons were performed using the Wilcoxon signed-rank test; differences in central tendency were expressed as Hodges–Lehmann median difference estimates, and effect sizes were reported via the rank-biserial correlation coefficient (*r_B_*).

To examine the temporal dynamics of autonomic regulation and HRV parameters, as well as Condition × Phase interaction effects, Linear Mixed Models (LMMs) were implemented. A 2 × 3 factorial design [Condition: Exercise vs. Control × Phase: Initial 10 min Intervention/Control, Equilibrio, 2-Back] was modeled for time-domain indices and geometric parameters, whereas a 2 × 2 factorial LMM [Condition × Phase: Protocol, 2-Back] was specified for frequency-domain spectral metrics. Participant ID was entered as a random intercept to account for within-subject dependency, and degrees of freedom for fixed effects were estimated using Satterthwaite’s approximation. Following statistically significant main or interaction effects, post hoc pairwise comparisons were adjusted for Type I error inflation using the Holm correction (*p_Holm_*) The threshold for statistical significance was set a priori at *p* < .05.

## 3. Results

### 3.1. Anthropometric and Demographic Characteristics

A total of *n* = 47 healthy volunteers (23 males, 24 females) aged 18 to 29 years successfully completed the study. The Shapiro–Wilk normality test results for demographic and anthropometric parameters indicated that height and body weight followed a normal distribution (*p* > .05). In contrast, age across the total sample, BMI values among female participants, and IPAQ scores among male participants deviated significantly from normality (***p*** < .05). Descriptive statistics and normality distribution metrics, stratified by sex, are presented in detail in Table 1.

### 3.2. Exercise Protocol and Physiological Manipulation Check

During the 10 min treadmill protocol at a constant speed of 8 km/h, participants covered a standardized distance of 1.33 km. The acute aerobic exercise intervention elicited substantial cardiovascular strain, as evidenced by a significantly elevated mean heart rate throughout the exercise protocol (146.90 ± 20.78 bpm; corresponding to ~74% of age-predicted HR_max_) compared to the time-matched passive control condition (79.54 ± 6.69 bpm; ***p*** < .001). This marked cardiovascular response confirms that the experimental intervention successfully induced the intended moderate-intensity aerobic exercise stimulus prior to cognitive testing.

Additionally, Borg RPE scores significantly increased from pre-exercise baseline (7.13 ± 1.48) to immediately post-exercise (11.30 ± 3.56; ***W*** = 4.00, ***z*** = −5.27, ***p*** < .001, ***r_B_*** = −0.991). The full distribution of RPE scores was: pre-exercise median = 6.0 (IQR: 6.0–8.0; range: 6–10) and post-exercise median = 10.0 (IQR: 9.0–14.0; range: 5–20).

### 3.3. Motor–Cognitive Task Outcomes

Regarding performance on the motor–cognitive *Equilibrio* task, completion times differed significantly between the exercise condition (81.36 ± 27.92 s) and the control condition (89.11 ± 23.15 s; ***W*** = 292.5, ***z*** = −2.873, ***p*** = .004, ***r_B_*** = −0.481). This finding indicates that following acute submaximal exercise stimulation, participants completed the visuospatial–motor task significantly faster compared to the resting control condition (median Hodges–Lehmann difference: −9.50 s).

### 3.4. Cognitive Performance Outcomes

Participants’ working memory and sustained attention performance were evaluated across response time dynamics (mean reaction time [Mean RT], reaction time variability [SDRT], and the intra-individual coefficient of variation [CV]), target detection and error metrics (hits, false alarms, misses), and Signal Detection Theory (SDT) parameters (perceptual sensitivity [*d*′] and response bias [*c*]):

#### 3.4.1. Reaction Time and Variability Dynamics (Parametric Analysis)

Paired-samples *t*-tests conducted on normally distributed reaction time metrics revealed no statistically significant differences between the exercise and control conditions for mean reaction time (***t***(46) = −0.133, ***p*** = .895), standard deviation of reaction times (SDRT; ***t***(46) = −0.246, ***p*** = .807), or intra-individual coefficient of variation (CV; ***t***(46) = −0.402, ***p*** = .689).

#### 3.4.2. Accuracy, Target Detection, and Error Rates (Non-Parametric Analysis)

Wilcoxon signed-rank tests applied to non-normally distributed accuracy and error scores indicated no significant differentiation between conditions in terms of correct target hits (***W*** = 421.5, ***z*** = −0.622, ***p*** = .536, ***r_B_*** = −0.109), hit rate (***W*** = 400.5, ***z*** = −0.875, ***p*** = .384, ***r_B_*** = −0.153), or missed targets (***W*** = 524.5, ***z*** = 0.622, ***p*** = .536, ***r_B_*** = 0.109). Similarly, false alarm rates (***W*** = 392.0, ***z*** = −0.242, ***p*** = .813, ***r_B_*** = −0.044) and overall accuracy percentages (***W*** = 504.5, ***z*** = 0.111, ***p*** = .916, ***r_B_*** = 0.019) remained virtually identical across both experimental conditions.

#### 3.4.3. Signal Detection Theory (SDT) Metrics (*d*′ and *c*)

##### Perceptual Sensitivity Index (*d*′)

The perceptual sensitivity index (*d*′), reflecting participants’ capacity to discriminate target signals from background noise, exhibited no statistically significant difference between the exercise (1.655 ± 0.580) and control (1.696 ± 0.583) conditions (***W*** = 501.5, ***z*** = −0.426, ***p*** = .674, ***r_B_*** = −0.072).

##### Response Bias/Decision Criterion (*c*)

The response bias criterion (*c*), denoting decision-making strategy, remained comparably conservative and negative across both exercise (−1.619 ± 0.512) and control (−1.642 ± 0.551) conditions, yielding no statistically significant difference (***W*** = 564.0, ***z*** = 0.257, ***p*** = .802, ***r_B_*** = 0.043).

### 3.5. Time-Domain and Frequency-Domain Autonomic Indicators

Analysis of autonomic regulatory dynamics across experimental phases (Protocol: Exercise vs. Control, *Equilibrio* task, and 2-Back task) demonstrated that the acute exercise condition induced pronounced parasympathetic withdrawal—evidenced by marked reductions in RMSSD, SDNN, PNS Index, and HF spectral power—alongside significant elevations in sympathetic modulation (SNS Index, LF/HF ratio) and cardiovascular strain (Stress Index) across all evaluation time points.

Autonomic stress metrics peaked during the motor–cognitive game phase. Although a progressive trend toward parasympathetic reactivation emerged during the subsequent 2-Back working memory task, vagal markers remained significantly suppressed relative to the control condition throughout cognitive phasing. Pairwise non-parametric condition comparisons, descriptive statistics, and corresponding effect sizes across all experimental phases are detailed in Table 2.

### 3.6. Linear Mixed Model (LMM) Evaluation of Autonomic Modulation Dynamics

To track the temporal trajectories of heart rate variability and autonomic indices across experimental phases, Linear Mixed Models (LMMs) were implemented, revealing statistically significant Condition × Phase interactions across all time-domain metrics and autonomic indices (*p* < .05; Table 3).

Parasympathetic markers (RMSSD and PNS Index) reached nadir levels during both the acute exercise intervention and the subsequent motor–cognitive game phases, indicating profound vagal withdrawal. During the 2-Back working memory task, although the exercise condition exhibited a significant vagal reactivation compared to the game phase (20.31 ± 2.55 ms; ***p_Holm_*** < 0.001), parasympathetic tone failed to return to baseline resting levels of the control condition (34.16 ± 2.55 ms) and remained significantly suppressed (***p_Holm_*** < 0.001).

In parallel, cardiovascular stress load peaked during the post-exercise motor task (50.17 ± 2.14) and remained markedly elevated during the 2-Back phase (25.52 ± 2.14) relative to the control condition (11.02 ± 2.14), confirming sustained sympathetic activation (***p_Holm_*** < 0.001).

In the frequency domain, a significant Condition x Phase interaction was detected for the LF/HF ratio (***F***(1138) = 10.11, ***p*** = .002). The sympathovagal ratio during the cognitive task remained substantially higher in the exercise condition (6.671 ± 0.564) compared to the control condition (2.307 ± 0.564; ***p_Holm_*** < 0.001), confirming persistent sympathetic dominance during cognitive phasing.

## 4. Discussion

The present study investigated the effects of acute aerobic exercise on sequentially administered cognitive tasks—namely, the visuospatial construction-based *Equilibrio* task and the working memory-based 2-Back task—alongside underlying autonomic nervous system dynamics. The primary finding of this investigation reveals that the cognitive benefits of acute aerobic exercise are domain-specific and do not transfer to a subsequent, qualitatively distinct cognitive task, despite sustained physiological autonomic activation.

The acute exercise intervention significantly reduced completion time in the *Equilibrio* task compared to the control condition (***W*** = 292.5, ***z*** = −2.873, ***p*** = .004, ***r_B_*** = −0.481). In contrast, no statistically significant differences were observed across any behavioral performance dimensions in the immediately following 2-Back task. This dissociation aligns with recent evidence demonstrating that acute exercise selectively enhances the visuospatial subcomponents of working memory while leaving phonological and analytical processing unaffected (Borrega-Alonso & Otamendi, 2025). Our findings provide a novel contribution to the existing literature by directly testing this domain-specific dissociation within the same cohort using a sequential multi-task paradigm.

This response pattern is consistent with recent findings in occupational contexts; Agostinelli et al. (2025) reported that acute aerobic or resistance exercise exerted no discernible effect on cognitive flexibility during subsequent simulated occupational firefighter tasks, despite inducing robust alterations in cardiac autonomic regulation (HRV). This distinct “autonomic shift without behavioral change” response closely mirrors our observations and suggests that the dissociation between sustained autonomic modulation and executive task performance is a systematic rather than incidental phenomenon.

A notable finding of the present study is the clear dissociation observed between autonomic nervous system responses and behavioral cognitive performance. During the 2-Back task following the exercise condition, sympathetic activity markers increased markedly, whereas parasympathetic/vagal modulation indices were significantly suppressed; however, this sustained autonomic activation failed to alter behavioral performance.

This finding can be evaluated within the framework of the transient hypofrontality hypothesis, which is widely utilized to explain the exercise–cognition relationship. According to this hypothesis, while exercise elevates neural activity in motor, sensory, and autonomic pathways, it induces a transient downregulation in prefrontal structures governing executive functions due to competitive metabolic resource allocation (Dietrich, 2006). In line with this theoretical framework, endurance exercise has been shown to selectively impair prefrontal cortex–dependent (executive function) tasks while preserving other cognitive processes (Dietrich & Sparling, 2004). Nevertheless, it should be emphasized that such detrimental effects primarily manifest during ongoing exercise and within dual-task paradigms (Szameitat et al., 2016). In support of this distinction, a meta-analysis synthesizing 22 empirical studies revealed that high-intensity exercise impairs exclusively concurrent prefrontal-dependent tasks, whereas low-to-moderate-intensity protocols induce no such impairment (Jung et al., 2022).

Because our experimental design administered cognitive assessments immediately post-exercise rather than concurrently, the absence of behavioral changes despite substantial autonomic shifts (sympathetic dominance, vagal withdrawal) may be consistent with the possibility that transient hypofrontality effects had largely resolved upon exercise cessation. Interpreted alongside the neurovisceral integration model—which posits that prefrontal recruitment bolsters parasympathetic tone, dampens sympathetic outflow, and directly modulates HRV metrics (Nikolin et al., 2017)—our observed pattern of sympathetic dominance and vagal withdrawal would be consistent with temporarily attenuated prefrontal regulation that nonetheless remained sufficient to preserve 2-Back task performance without behavioral impairment. It should be emphasized that no EEG, fNIRS, fMRI, cerebral blood-flow, neurochemical, or other cortical physiological measure was obtained in this study, and HRV alone cannot directly establish the state of prefrontal activation. The interpretations above are therefore offered as plausible explanations consistent with the observed autonomic–behavioral dissociation, and not as mechanisms directly demonstrated by the present data.

Furthermore, our findings are not fully consistent with models explaining the cognitive effects of acute exercise solely through generalized physiological arousal or unidirectional resource depletion; had the observed outcomes stemmed merely from systemic physiological arousal, heightened sympathetic activation would have been expected to modulate 2-Back performance in some direction. Instead, our data support the premise that the physiological and cognitive sequelae of acute exercise can dissociate and proceed along independent trajectories. It should be noted, however, that the present study did not independently manipulate or measure general physiological arousal, and the control condition differed from the exercise condition in several physiological and psychological respects beyond arousal per se. The present findings are therefore inconsistent with a simple account in which generalized physiological activation necessarily improves performance on all subsequent cognitive tasks, but they do not rule out contributions from general arousal, motivation, fatigue, or other systemic mechanisms. Rather than definitively refuting general-arousal explanations, our results suggest that the cognitive effects of acute exercise may depend on task characteristics, timing, and exercise dose.

These observations correspond with the existing literature indicating that cognitive task timing during the acute post-exercise window governs performance outcomes, while further suggesting that task modality—rather than strictly the post-exercise delay—serves as a critical determinant. Prior meta-analytic evidence indicates that acute moderate-intensity exercise selectively enhances distinct subcomponents of executive function across diverse age and fitness cohorts (Ludyga et al., 2016). By directly testing this domain specificity through sequentially administered tasks within the same participants, our study contributes to addressing this empirical gap: the cognitive facilitation remained confined to the task temporally closest to exercise cessation that engaged the visuospatial domain, without generalizing to the subsequent task targeting an alternative executive subcomponent.

Regarding exercise duration and intensity within the dose–response literature, previous investigations in adults demonstrate that a 20 min bout of moderate-to-vigorous exercise significantly improves inhibitory control, whereas a 10 min bout at equivalent intensity fails to elicit comparable benefits (Chang et al., 2019). Consequently, the 10 min aerobic protocol employed in our study may have fallen below the stimulus threshold required to generate measurable gains in an executive-heavy working memory paradigm such as the 2-Back task. This indicates that the observed task-specific pattern may stem not only from cognitive domain distinctions, but also from divergent sensitivity thresholds across cognitive tasks in response to exercise dosage. Although the marked enhancement in the *Equilibrio* task suggests that visuospatial and fine-motor components may be more sensitive to shorter exercise durations, future investigations should systematically compare varying exercise durations (e.g., 10, 20, and 30 min) to directly determine dose–response interactions.

### Limitations and Directions for Future Research

Several limitations of the present study warrant consideration. First, the sequential presentation of cognitive tasks was fixed (*Equilibrio* followed by 2-Back) rather than counterbalanced, which represents a central limitation affecting the primary conclusions rather than a minor caveat. Because task order was fixed, task type is confounded with the time elapsed since exercise cessation, cumulative physiological and/or mental fatigue, possible carry-over effects from the first task, fluctuations in motivation or alertness, and differences in task duration and cognitive load between the two tasks. Accordingly, the present findings should be interpreted as describing the pattern of results observed within this specific fixed-order, sequential paradigm, rather than as evidence of a generalizable, order-independent dissociation between visuospatial–motor and executive working memory sensitivity to acute exercise. Although randomized condition allocation and extensive familiarization protocols substantially mitigated practice biases, future investigations should implement fully counterbalanced task orders to validate the generalizability of this sequential profile. Second, the absence of direct neuroimaging or cortical electrophysiological modalities (e.g., EEG or fNIRS) renders our mechanistic inferences regarding transient hypofrontality indirect. Future research coupling simultaneous neuroimaging with autonomic HRV telemetry is recommended to elucidate the central neurovascular mechanisms mediating this autonomic–cognitive dissociation. Third, the *Equilibrio* task is a composite visuospatial–motor construction task that recruits visuospatial processing, mental manipulation/simulation, motor planning, fine motor coordination, and hand–eye coordination; consequently, the significant reduction in completion time following exercise cannot be attributed uniquely to enhanced visuospatial working memory, and completion time alone cannot dissociate contributions from motor speed, planning efficiency, visuospatial reasoning, or general arousal. In the present study, task performance was indexed solely by completion time; construction accuracy or error rates were not scored, precluding assessment of whether faster completion following exercise was achieved without any compromise in structural accuracy. Future studies should incorporate additional outcome measures (e.g., construction accuracy and movement kinematics) to better isolate these component processes. Fourth, although the fixed absolute treadmill speed (8 km/h) elicited a significantly elevated heart rate relative to the control condition, confirming that a moderate-intensity exercise stimulus was achieved on average across the sample, this absolute workload was not individualized to participants’ fitness levels; the same absolute speed may therefore have corresponded to different relative exercise intensities across participants. Fifth, respiratory rate and depth, which can substantially influence HRV independent of autonomic tone, were not measured or controlled in the present study. Finally, the cohort was composed exclusively of young, healthy undergraduate students, which limits the ecological generalizability of these findings across diverse clinical populations, older demographics, varied physical fitness strata, and alternative exercise modalities.

## 5. Conclusions

In conclusion, within this fixed-order, sequential paradigm, our findings suggest that the cognitive benefits of acute aerobic exercise manifest in a task-specific manner that operates independently of sustained autonomic arousal, failing to transfer to an immediately subsequent task engaging a distinct executive subcomponent. These results suggest that the complex interplay governing exercise–cognition interactions cannot be fully captured by broad physiological arousal or unidirectional transient hypofrontality frameworks alone, but rather necessitates a domain-specific and temporally sensitive theoretical perspective. Given the fixed task order and the absence of direct neural measures, these conclusions should be regarded as hypothesis-generating and specific to the present paradigm, warranting confirmation in future counterbalanced designs incorporating neuroimaging.

## Figures and Tables

**Table 1:** Participant Demographics and Anthropometric Characteristics Stratified by Gender (N = 47).

Parameter	Gender	Mean ± SD	Min–Max	Shapiro–Wilk (*W*)	*p*
**Age (year)**	Male	21.30 ± 3.10	18.00–29.00	0.850	.003
**Age (year)**	Female	20.79 ± 2.21	18.00–27.00	0.851	.002
**Height (cm)**	Male	180.0 ± 5.22	170.0–189.0	0.972	.737
**Height (cm)**	Female	166.8 ± 5.57	157.0–176.0	0.965	.535
**Body Weight (kg)**	Male	78.35 ± 9.14	61.00–100.0	0.973	.771
**Body Weight (kg)**	Female	61.58 ± 8.38	50.00–82.00	0.929	.091
**BMI (kg/m^2^)**	Male	24.15 ± 2.22	20.62–29.86	0.950	.295
**BMI (kg/m^2^)**	Female	21.95 ± 2.91	17.76–30.43	0.839	.001
**IPAQ Total MET (min/week)**	Male	4532 ± 4290	119.0–17,230	0.792	<.001
**IPAQ Total MET (min/week)**	Female	3214 ± 1939	372.0–7200	0.943	.195

**Note:** SD = Standard Deviation, Min = Minimum, Max = Maximum, BMI = Body Mass Index, IPAQ = International Physical Activity Questionnaire.

**Table 2:** Paired Non-Parametric Comparisons of Autonomic and HRV Parameters Between Exercise and Control Conditions (N = 47).

Parameter	Exercise	Control	*W*	*z*	*p*	Hodges–Lehmann	Rank Biserial (*r_B_*)
***Protocol Phase***							
**Mean RR (ms)**	417.1 ± 60.09	759.5 ± 60.73	0.000	−5.968	**<.001**	−342.80	−1.000
**Mean HR (bpm)**	146.9 ± 20.78	79.54 ± 6.69	1128.0	5.968	**<.001**	67.11	1.000
**RMSSD (ms)**	11.50 ± 16.79	37.92 ± 16.95	82.00	−5.101	**<.001**	−27.66	−0.855
**SDNN (ms)**	13.19 ± 13.69	47.24 ± 16.00	35.00	−5.598	**<.001**	−34.65	−0.938
**PNS Index**	−3.67 ± 0.75	−0.84 ± 0.78	2.000	−5.947	**<.001**	−2.880	−0.996
**SNS Index**	10.62 ± 5.18	0.88 ± 0.87	1128.0	5.968	**<.001**	9.022	1.000
**Stress Index**	31.62 ± 15.22	9.63 ± 3.18	1125.0	5.937	**<.001**	19.68	0.995
**HF_log_**	4.85 ± 1.76	6.02 ± 1.21	278.0	−2.868	**.004**	−1.100	−0.486
**LF_log_**	4.07 ± 2.03	7.35 ± 0.68	24.00	−5.714	**<.001**	−3.285	−0.957
**HF_n.u._**	9.23 ± 12.5	32.44 ± 9.67	45.00	−5.492	**<.001**	−24.65	−0.920
**LF_n.u._**	13.18 ± 21.42	68.05 ± 10.37	8.000	−5.884	**<.001**	−59.26	−0.986
**LF/HF Ratio**	3.74 ± 2.3	2.85 ± 1.97	783.0	2.317	**.021**	1.036	0.388
***Equilibrio Task Phase***							
**Mean RR (ms)**	460.0 ± 71.16	729.0 ± 60.70	1.000	−5.958	**<.001**	−266.30	−0.998
**HR (bpm)**	134.0 ± 22.26	82.92 ± 7.33	1127.0	5.958	**<.001**	49.31	0.998
**RMSSD (ms)**	7.69 ± 6.08	30.24 ± 12.18	26.00	−5.693	**<.001**	−21.81	−0.954
**SDNN (ms)**	10.81 ± 7.37	39.30 ± 11.68	1.000	−5.958	**<.001**	−27.97	−0.998
**PNS Index**	−3.58 ± 0.70	−1.20 ± 0.66	0.000	−5.968	**<.001**	−2.318	−1.000
**SNS Index**	12.15 ± 7.29	1.50 ± 1.14	1127.0	5.958	**<.001**	10.26	0.998
**Stress Index**	50.17 ± 29.36	12.19 ± 4.29	1110.0	5.778	**<.001**	34.27	0.968
***2-Back Task Phase***							
**Mean RR (ms)**	538.50 ± 77.40	756.1 ± 66.63	0.000	−5.968	**<.001**	−213.80	−1.000
**HR (bpm)**	114.00 ± 17.41	80.01 ± 7.39	1128.0	5.968	**<.001**	32.03	1.000
**RMSSD (ms)**	20.31 ± 29.63	34.16 ± 14.23	100.0	−4.910	**<.001**	−17.52	−0.823
**SDNN (ms)**	25.05 ± 22.56	41.17 ± 13.10	82.00	−5.101	**<.001**	−18.50	−0.855
**PNS Index**	−2.65 ± 0.72	−0.91 ± 0.78	0.000	−5.968	**<.001**	−1.705	−1.000
**SNS Index**	6.21 ± 3.63	1.11 ± 1.03	1115.0	5.831	**<.001**	4.280	0.977
**Stress Index**	25.52 ± 12.49	11.02 ± 3.40	1104.0	5.714	**<.001**	12.91	0.957
**HF_log_**	3.41 ± 1.56	5.72 ± 0.90	27.00	−5.683	**<.001**	−2.285	−0.952
**LF_log_**	3.98 ± 1.53	6.58 ± 0.46	0.000	−5.905	**<.001**	−2.630	−1.000
**HF_n.u._**	7.76 ± 8.98	32.39 ± 10.18	11.00	−5.852	**<.001**	−25.65	−0.980
**LF_n.u._**	37.24 ± 19.41	67.64 ± 10.19	39.00	−5.556	**<.001**	−32.12	−0.931
**LF/HF Ratio**	6.67 ± 6.92	2.30 ± 1.63	1060.0	5.249	**<.001**	2.903	0.879

**Note:** M = Mean; SD = Standard Deviation; ms = milliseconds; PNS = Parasympathetic Nervous System; SNS = Sympathetic Nervous System; n.u. = normalized units; *W* = Wilcoxon test statistic; *z* = standardized test statistic; *r_B_* = Rank-Biserial correlation effect size. The Equilibrio motor task phase was excluded from frequency-domain spectral analysis due to methodological requirements regarding non-stationary signal constraints and minimal short-term recording duration guidelines.

**Table 3:** ANOVA and Marginal Mean Results for the Linear Mixed Model (LMM) Examining the Effects of Conditions and Phases on Autonomous Modulation (N = 47).

Parameter	Model Design	*F*	*df*	*p*	Protocol [Exercise vs. Control]	Equilibrio Game [Exercise vs. Control]	2-Back Task [Exercise vs. Control]
**Mean RR (ms)**	2 × 3	26.22	2230	**<.001**	417. 10 ± 9.69 vs. 759.50 ± 9.69	460.00 ± 9.69 vs. 729.00 ± 9.69	538.50 ± 9.69 vs. 756.10 ± 9.69
**Mean HR (bpm)**	2 × 3	32.27	2230	**<.001**	146.90 ± 2.22 vs. 79.54 ± 2.22	134.00 ± 2.22 vs. 82.92 ± 2.22	114.00 ± 2.22 vs. 80.01 ± 2.22
**RMSSD (ms)**	2 × 3	3.469	2230	**.033**	11.50 ± 2.55 vs. 37.92 ± 2.55	7.69 ± 2.55 vs. 30.24 ± 2.55	20.31 ± 2.55 vs. 34.16 ± 2.55
**SDNN (ms)**	2 × 3	10.16	2230	**<.001**	13.19 ± 2.16 vs. 47.24 ± 2.16	10.81 ± 2.16 vs. 39.30 ± 2.16	25.05 ± 2.16 vs. 41.17 ± 2.16
**PNS Index**	2 × 3	16.04	2230	**<.001**	−3.678 ± 0.11 vs. −0.845 ± 0.11	−3.584 ± 0.11 vs. −1.201 ± 0.11	−2.654± 0.11 vs. −0.913 ± 0.11
**SNS Index**	2 × 3	14.49	2230	**<.001**	10.62 ± 0.58 vs. 0.88± 0.58	12.15 ± 0.58 vs. 1.49 ± 0.58	6.20 ± 0.58 vs. 1.10 ± 0.58
**Stress Index**	2 × 3	17.39	2230	**<.001**	31.62 ± 2.14 vs. 9.634 ± 2.14	50.17 ± 2.14 vs. 12.19 ± 2.14	25.52 ± 2.14 vs. 11.02 ± 2.14
**HF_log_ (ms^2^)**	2 × 2	7.787	1184	**.006**	4.85 ± 0.20 vs. 6.02 ± 0.20	—	3.41 ± 0.20 vs. 5.72 ± 0.20
**LF_log_ (ms^2^)**	2 × 2	3.219	1138	**.075**	4.07 ± 0.20 vs. 7.35 ± 0.20	—	3.98 ± 0.20 vs. 6.58 ± 0.20
**HF_n.u._ (ms^2^)**	2 × 2	0.320	1138	**.573**	9.23 ± 1.52 vs. 32.44 ± 1.52	—	7.76 ± 1.52 vs. 32.39 ± 1.52
**LF_n.u._ (ms^2^)**	2 × 2	40.04	1138	**<.001**	13.18 ± 2.36 vs. 68.05 ± 2.36	—	37.24 ± 2.36 vs. 67.64 ± 2.36
**LF/HF Ratio**	2 × 2	10.11	1138	**.002**	3.74 ± 0.56 vs. 2.85 ± 0.56	—	6.67 ± 0.56 vs. 2.30 ± 0.56

**Note:** The F and df values reported in the table reflect the Condition × Phases interaction effects in Linear Mixed Models (LMM). Values are presented as Estimated Marginal Mean ± Standard Error. Statistical parameters reflect the results of a Type III Sum of Squares ANOVA, for which the Satterthwaite approximation was used for degrees of freedom (df). Participant_ID was modeled as a random interaction. ms = milliseconds; bpm = beats per minute; PNS = Parasympathetic Nervous System; SNS = Sympathetic Nervous System; n.u. = normalized units; HFlog/LFlog = natural logarithms of spectral powers. The motor equilibrium task phase was excluded from the frequency-domain spectral analyses due to non-stationarity and short recording duration.

## Data Availability

The datasets used and/or analyzed during the current study are available from the corresponding author upon reasonable request.
